# Impact of Basic Course in Biomedical Research (BCBR) on Medical Teachers and Postgraduate Students Across India: A Cross-Sectional Survey-Based Study

**DOI:** 10.7759/cureus.62908

**Published:** 2024-06-22

**Authors:** Krupal J Joshi, Sanjay Singhal, Sanjay Gupta

**Affiliations:** 1 Community and Family Medicine, All India Institute of Medical Sciences, Rajkot, Rajkot, IND; 2 Pulmonary Medicine, Tribeni Sahai (T.S) Misra Medical College and Hospital, Lucknow, IND; 3 Forensic Medicine and Toxicology, All India Institute of Medical Sciences, Rajkot, Rajkot, IND

**Keywords:** medical teachers and post-graduate students, basic course in bio-medical research, research skills, medical education, biomedical research

## Abstract

Introduction

Despite world-class medical facilities and clinical expertise in India, various challenges have hampered biomedical research, including limited funding, overburdened healthcare professionals, and inadequate research infrastructure. The National Medical Commission (NMC) has recognized the need to enhance research capabilities in the medical community and mandated the Basic Course in Biomedical Research (BCBR). This study evaluates the impact of BCBR on medical teachers and postgraduate students across India.

Methods

A cross-sectional survey-based study was conducted among 245 participants who completed BCBR, out of 1,187 who were sent the questionnaire. A structured questionnaire collected data on demographics, motivations for taking the course, knowledge and skills improvement, and research output. Statistical analyses were performed using IBM SPSS Statistics for Windows, Version 29.0 (Released 2022; IBM Corp., Armonk, NY, USA).

Results

Participants included diverse age groups, with motivations ranging from regulatory compliance to a genuine desire for research knowledge. The majority scored over 70% in the course, indicating substantial knowledge improvement. BCBR positively influenced research output with increased research proposal submissions, publications, and improved thesis mentoring. The analysis showed that participants’ designations and branches of study significantly affected course performance, while gender did not. The study revealed a strong correlation between pre-course and post-course performance across various research topics.

Conclusion

BCBR, mandated by the NMC, not only fulfills regulatory requirements but also promotes a research culture in India’s medical community.

## Introduction

Medical research is pivotal in advancing healthcare knowledge and refining treatment strategies. Despite India’s world-class medical facilities and a rich history of producing eminent researchers, the research landscape faces significant challenges, including limited funding, overburdened medical professionals, and inadequate research infrastructure [1]. Only a small fraction of Indian institutions publish many research papers, lagging far behind their international counterparts [2]. Recognizing the need for improvement, the National Medical Commission (NMC) in India has mandated that postgraduate medical students undertake original research as part of their theses and publish research papers to qualify for appointments and promotions within medical colleges and research institutions [3]. However, due to variations in infrastructure and the heterogeneous training of medical students, many need more proficiency in basic health research methodologies. To address this gap, the NMC has mandated a standardized research methodology course, the Basic Course in Biomedical Research (BCBR), offered by the Indian Council of Medical Research-National Institute of Epidemiology in Chennai [4,5]. This online course encompasses 23 modules delivered through video lectures, supported by reading materials, assignments, and a final proctored examination [5]. Successful course completion, with a 50% or higher score, results in an e-verifiable course completion certificate [5]. Given that the BCBR course is now a prerequisite for postgraduate degrees, recruitment, and promotions in medical teaching, this study aims to assess its impact on enhancing research skills beyond meeting regulatory requirements.

## Materials and methods

This cross-sectional observational study was performed at the All India Institute of Medical Sciences (AIIMS), Rajkot, India. Ethical approval was obtained from the Institutional Ethics Committee (IEC) of AIIMS, Rajkot (AIIMS.Rajkot/IEC/17/2022).

This study included participants from medical institutes across India who had undergone the BCBR course, irrespective of their designation and marks in the BCBR course. Data was collected through a newly developed, structured, and validated questionnaire distributed via Google Forms. The questionnaire was validated by the involvement of experts in the field (biostatisticians, epidemiologists, and clinical researchers), and a pilot sampling was carried out to correct ambiguities. The data collected from the pilot sampling were not included in the final analysis. Informed consent was obtained from all the participants before the collection of data. The questionnaire gathered participants’ general information (age, gender, and designation), qualifications (graduate or postgraduate degree), additional degrees in research methodology, prior involvement in research or ethical boards, motivations for pursuing the BCBR course, and its impact on knowledge (overall marks in the course), skills (assessed all the 18 topics of BCBR based on Likert scale of 5 points ranging from “strongly agree” to “strongly disagree” before and after this course), and research output (number of publications in indexed journal, the requirement of statistician, number of the research proposal, assistance of postgraduate thesis and enhancement of research culture). The Google Forms questionnaire link was circulated to all concerned and relevant groups in various medical institutes in India.

Sample size and statistical analysis

According to the pilot study results, 20% of postgraduate students and medical teachers had completed the BCBR. To calculate the required sample size, we used the formula N = t2.p.q/d2, where n represents the sample size, t was equivalent to the confidence level at 95% (standard value of 1.96), p was equal to the estimated prevalence of postgraduate students and medical teachers who had completed BCBR, q was equal to 1 minus the prevalence, and d was the absolute precision (taken as 5%; 0.05). Based on the anticipated prevalence of 20%, our sample size was determined to be 245.86 (rounded up to 245).

Data was collected and analyzed using IBM SPSS Statistics for Windows, Version 20.0 (Released 2011; IBM Corp., Armonk, NY, USA). After analyzing the data for normality, the data were represented as mean, SD, 95% CI, and median for quantitative data analysis. Nonparametric data were analyzed through Mann-Whitney U/unpaired t-tests, and parametric data through Wilcoxon signed-rank/paired t-tests. A p-value of ≤0.05 was considered statistically significant.

## Results

Participant demographics and motivation

Out of 1,187 individuals who were sent the questionnaire, the study included 245 participants who completed it. The study represents a diverse demographic profile, with ages 31-40 years in 78 (32%) and males in 151 (61.63%). Professionally, professors constituted the largest group (77, 31.4%), and many participants held additional qualifications related to research, particularly the Revised Basic Course Workshop (124, 50.6%) (Table 1).

**Table 1:** Demographic and professional attributes of participants who pursued BCBR BCBR, Basic Course in Biomedical Research; NMC, National Medical Commission

Particulars	Number (%), total n = 245
Age range	
20-30	54 (22%)
31-40	78 (32%)
41-50	59 (24%)
51-60	52 (21%)
61-70	2 (1%)
Gender	
Male	151 (61.63%)
Female	94 (38.37%)
Designation	
Professor	77 (31.4%)
Additional professor	4 (1.6%)
Associate professor	41 (16.7%)
Assistant professor	66 (26.9%)
Senior resident/tutor	21 (8.6%)
Junior resident	36 (14.7%)
Additional qualifications or experience in medical education and research	
Advanced course in medical education	64 (26%)
Attitude and communication	2 (0.8%)
Bioethics	5 (2%)
Curriculum implementation support program	21 (8.6%)
Research methodology course	22 (9%)
Good clinical practice	7 (2.9%)
Revised basic course workshop	124 (50.6%)
Reason for pursuing the BCBR course	
For carrying out research	2 (0.8%)
For knowledge purpose	17 (6.9%)
Mandatory by NMC	130 (53.1%)
All of the above	96 (39.2%)

**Figure 1:**
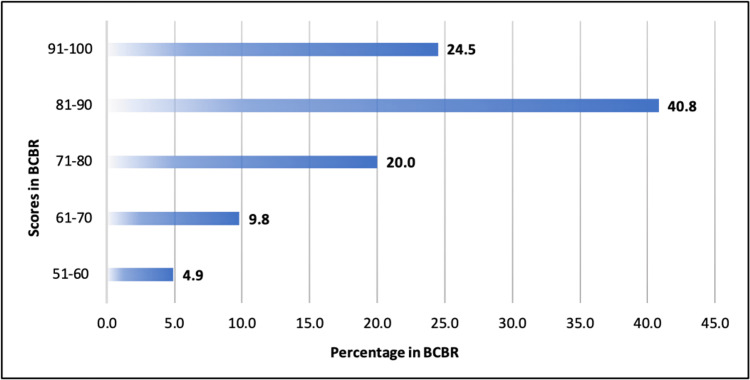
Distribution of scores in the final BCBR evaluation BCBR, Basic Course in Biomedical Research

Participants’ motivations for pursuing the BCBR course were multifaceted, with 130 (53.1%) driven by the NMC’s mandatory requirement, 96 (39.2%) citing a combination of factors, and smaller percentages pursuing the course solely for knowledge acquisition (17, 6.9%) or research (2, 0.8%). Most participants scored more than 209 (70%) in their final BCBR evaluations (Figure 1).

Impact on research output

The impact of the BCBR course on research output was noteworthy. The majority (147, 60%) reported that it facilitated the submission of more research proposals to the IEC. Similarly, a substantial proportion (148, 60.4%) credited the course with enhancing the publication of their research projects in reputable journals recognized by the NMC. Moreover, 163 (65.3%) of participants expressed that the BCBR course better equipped them to assist postgraduate residents with their theses and to instill a research-oriented culture within their departments and institutes. Nevertheless, a significant majority (145, 59.2%) acknowledged the continued need for assistance from statisticians, citing statistical analysis as a challenge (Table 2).

**Table 2:** Impact on research-related activities after pursuing a BCBR BCBR, Basic Course in Biomedical Research; IEC, Institutional Ethics Committee; NMC, National Medical Commission

Questions	Response		
	Yes	No	NA
Does the BCBR course help you submit more research proposals to the IEC?	147 (60%)	98 (40%)	-
Does the BCBR course help you in the publication of your research project in the Journal of Good Repute or NMC recognized?	148 (60.4%)	97 (39.6%)	-
Does BCBR better equip you to assist the thesis of your postgraduate resident effectively?	163 (65.3%)	51 (20.4%)	31 (12.2%)
Is BCBR better equipped to inculcate a research culture in the department or institute?	160 (65.3%)	85 (34.7%)	-
Are you still consulting statisticians to seek assistance in research work?	145 (59.2%)	100 (40.8%)	-

Effect of designation, branch of study, and gender

Statistical analyses were conducted to explore the influence of designation and branch of study on BCBR course performance. The Kruskal-Wallis test revealed no statistically significant difference in mean marks among various designations. This suggests that the designation may not significantly impact the marks obtained in the BCBR course (p = 0.24). However, regarding the branch of study (pre-clinical (anatomy, biochemistry, physiology, and pharmacology), para-clinical (pathology, microbiology, and radiology), and clinical (surgery, medicine, gynecology, pediatrics, pulmonary medicine, etc.)), a highly significant difference in mean marks was observed, indicating that the branch of study significantly influences BCBR course performance (p = 0.0001). An analysis of gender-based differences in BCBR course marks found no statistically significant distinction between male and female participants. The data exhibited a reasonably normal distribution (p = 0.11), and subsequent parametric unpaired t-tests yielded a nonsignificant result, with a t-value of 1.30 and a p-value of 0.19, suggesting no significant disparity in mean BCBR marks between male and female participants (Table 3).

**Table 3:** Association of variables and their impact on marks and publication

Variable	Mean score obtained	SD	Kolmogorov-Smirnov test/Shapiro-Wilk test/p-value	Mann-Whitney U test/unpaired t-test/Kruskal-Wallis test	p-value
Designation-wise marks distribution					
Professor	83.8	10.69	0.917/0.0001	1.399	0.24
Associate professor	84.6	6.83			
Assistant professor	82.7	9.17			
Senior resident	79.7	10.58			
Gender-wise marks distribution					
Female	83.5	9.91	1.146/0.11	1.308	0.19
Male	82.1	9.74			
Branch-wise marks distribution					
Pre-clinical	88.76	6.8	0.146/0.001	KW = 68.01	0.001
Para-clinical	80.11	9.1			
Clinical	72.78	10.5			
Number of articles published					
Before two years of course	3.48	4.83	0.811/0.001	U = 25576	0.0001
After two years of course	5.29	6.3			

Impact on publications and knowledge enhancement

A Mann-Whitney U test conducted to compare the number of publications before and after undertaking the BCBR course indicated a highly statistically significant difference in the number of publications between the two time periods (U = 25577, p = 0.0001). The mean scores for each course topic, both “Before” and “After” taking the course, indicated an average improvement in participants' understanding and knowledge of health research topics. This improvement was consistently observed across all topics, with statistical tests (paired t-tests or Wilcoxon signed-rank tests) always yielding highly significant p-values (all <0.001). These findings suggest that the improvements are unlikely due to random chance alone. Furthermore, correlation coefficients were calculated to assess the relationship between each course topic’s “Before” and “After” scores. Most topics exhibited positive correlations, indicating that individuals who performed well before the course tended to perform well afterward. These correlations ranged from 0.365 to 0.568, signifying moderate to strong positive associations between pre-course and post-course performance (Table 4).

**Table 4:** Impact of BCBR on various topics BCBR, Basic Course in Biomedical Research

		Mean	SD	Paired t-test/Wilcoxon signed-rank test	p-value	Correlation
Pair 1	Introduction to health research (Before)	2.79	1.095	13.635	0.001	0.466
	Introduction to health research (After)	3.74	1.003			
Pair 2	Formulating research questions, hypotheses, and objectives (Before)	2.8	1.135	14.734	0.001	0.365
	Formulating research question, hypothesis, and objectives (After)	3.9	0.991			
Pair 3	Literature review (Before)	2.8	1.14	14.879	0.001	0.407
	Literature review (After)	3.9	0.999			
Pair 4	Measures of disease frequency (Before)	2.82	1.237	16.382	0.001	0.502
	Measures of disease frequency (After)	3.9	0.995			
Pair 5	Qualitative research methods: an overview (Before)	2.48	1.126	15.684	0.001	0.409
	Qualitative research methods: an overview (After)	3.7	1.003			
Pair 6	Measurement of study variables (Before)	2.77	1.228	15.044	0.001	0.568
	Measurement of study variables (After)	3.82	0.972			
Pair 7	Sampling methods (Before)	2.79	1.213	14.876	0.001	0.568
	Sampling methods (After)	3.84	0.992			
Pair 8	Calculating sample size and power (Before)	2.64	1.271	15.547	0.001	0.441
	Calculating sample size and power (After)	3.76	1.091			
Pair 9	Validity of epidemiological studies (Before)	2.59	1.186	14.335	0.001	0.395
	Validity of epidemiological studies (After)	3.62	1.031			
Pair 10	Selection of study population (Before)	2.88	1.229	15.853	0.001	0.485
	Selection of study population (After)	3.96	0.966			
Pair 11	Study plan and project management (Before)	2.68	1.134	14.795	0.001	0.518
	Study plan and project management (After)	3.84	0.941			
Pair 12	Designing data collection tools (Before)	2.82	1.194	15.664	0.001	0.463
	Designing data collection tools (After)	3.88	0.98			
Pair 13	Principles of data collection (Before)	2.75	1.208	13.196	0.001	0.485
	Principles of data collection (After)	3.83	0.954			
Pair 14	Data management and analysis (Before)	2.83	1.248	14.812	0.001	0.061
	Data management and analysis (After)	3.82	0.963			
Pair 15	Ethical framework for health research (Before)	2.84	1.222	14.812	0.001	0.068
	Ethical framework for health research (After)	3.93	1.012			
Pair 16	Conducting clinical trials (if applicable) (Before)	2.47	1.187	10.624	0.001	0.078
	Conducting clinical trials (if applicable) (After)	3.36	1.236			
Pair 17	Prepare a protocol for research studies (Before)	2.91	1.247	13.319	0.001	0.544
	Prepare a protocol for research studies (After)	3.88	0.997			
Pair 18	Understanding and pursuance of publication ethics (including plagiarism) (Before)	2.84	1.186	13.42	0.001	0.509
	Understanding and pursuance of publication ethics (including plagiarism) (After)	3.88	1.053			

## Discussion

The present study reflects that participants with diverse perspectives and experiences undertook BCBR with a multifaceted motivation, including genuine enthusiasm for learning and research, supplemented with regulatory compliance mandated by the NMC. Our study also showed that BCBR helped in submitting more research proposals along with inculcating the research culture in their workplace, besides the ability to increase the number of research publications in reputed journals. All these findings support the idea that BCBR not only fulfills regulatory requirements but also promotes a research culture in India's medical community.

Research is helpful in the advancement of knowledge and the improvement of existing treatment strategies. Medicine and healthcare constantly evolve, and their current upgrade is only possible with research. However, in low- and middle-income countries (LMICs), which have 85% of the world’s population and 90% of the world’s health problems, only 10% of the global expenditure on health research is used [6]. Moreover, only 2% of the research publications in indexed journals are from LMICs [7]. A study about the status of research publications in India also showed that only 4.3% (25 out of 579) institutions published more than 100 papers per year, compared to 4,600 publications from the Massachusetts General Hospital [2]. Today, India is well known for its world-class medical facilities and depth of clinical expertise. We have a glorious past of producing great researchers in the field of medicine like Dr. Sambhu Nath De, Dr. Yellapragada Subbarow, Sir Upendra Nath Brahmachari, Dr. Subash Mukhopadhyay, Gopalasamudram Narayanan Ramachandran, and Obaid Siddiqi. With this glorious past and having clinical expertise with a vast population and healthcare needs, we have the potential to carry out outstanding biomedical research. A paucity of funds, overburdened physicians, and a lack of sophisticated research infrastructure are the most commonly cited reasons for this sorry affair of research in LMICs, including India [8]. A high-level committee by the Government of India recognized the importance of research and strongly recommended that medical teachers devote one-third of their time to research [9]. Other than these contributors, the lack of training in research methodology in medical institutes is a fundamental cause. To address the shortcomings of inadequate training in conducting research and encourage scientific inquiry and rational thinking, BCBR has been made mandatory by the NMC. Various studies have highlighted the potential benefits of short courses in biomedical research and biostatistics in improving the knowledge and skills of biomedical research [10-16]. Our analysis also documents a positive influence on knowledge as assessed by overall marks (scoring more than 70% in the majority) and skill, as evident in the positive difference between the “Before” and “After” BCBR with moderate to strong positive correlations ranging from 0.365 to 0.568. The findings of our study also suggest a positive influence of the BCBR course on participants' engagement in various research-related aspects, from proposal submission and publication to mentoring and fostering research culture. In practical terms, it suggests that participating in the BCBR course has substantially impacted individuals’ research productivity, as evidenced by a significant increase in publications following the course. This finding underscores the positive influence of the BCBR course on research output and highlights its effectiveness in enhancing research capabilities among participants. The main limitation of our study is that it assesses the participant’s skills and knowledge based only on the questionnaire. Moreover, we have assessed the impact of this course on all the participants as a single group, irrespective of qualifications and designations. Therefore, in the future, qualitative studies, along with real-time assessment, may be required to validate the findings of our studies.

## Conclusions

The BCBR, initiated by the NMC (formerly the Medical Council of India), is intended to build a research culture in India and has helped in achieving this goal through its positive impact on research capability and research output.
